# Brain network-based stratification of mental health disorders: design and cohort description of the STRATIFY and ESTRA studies

**DOI:** 10.1038/s41380-026-03779-x

**Published:** 2026-07-22

**Authors:** Nilakshi Vaidya, Zuo Zhang, Kofoworola Agunbiade, Georgina Keggin, Kebir Hedi, Jeanne Winterer, Marina Bobou, John Broulidakis, Sinead King, Lauren Robinson, Yuning Zhang, Gareth J. Barker, Arun L. Bokde, Rüdiger Brühl, Antoine Grigis, Hervé Lemaître, Tristam Lett, Frauke Nees, Dimitri Papadopoulos, Luise Poustka, Ulrike Schmidt, Julia Sinclair, Argyris Stringaris, Robert Whelan, Henrik Walter, Sylvane Desrivières, Gunter Schumann

**Affiliations:** 1 Centre for Population Neuroscience and Stratified Medicine (PONS), Department of Psychiatry and Neuroscience, Charité Universitätsmedizin Berlin, Berlin, Germany; 2 Social Genetic and Developmental Psychiatry Centre, Institute of Psychiatry, Psychology and Neuroscience, King’s College London, London, SE5 8AF United Kingdom; 3 School of Psychology, Institute for Mental Health, University of Birmingham, Birmingham, UK; 4 Department of Education and Psychology, Freie Universität Berlin, Berlin, Germany; 5 Charité – Universitätsmedizin Berlin, corporate member of Freie Universität Berlin, Humboldt-Universität zu Berlin, and Berlin Institute of Health, Department of Psychiatry and Psychotherapy, Campus Charité Mitte, Charitéplatz 1, Berlin, Germany; 6 Psychology Department, University of Southampton, Southampton, UK; 7 Oxford Institute of Clinical Psychology Training and Research, Oxford University, Oxford, UK; 8 Department of Neuroimaging, Institute of Psychiatry, Psychology & Neuroscience, King’s College London, London, SE5 8AF United Kingdom; 9 Discipline of Psychiatry, School of Medicine and Trinity College Institute of Neuroscience, Trinity College Dublin, Dublin, Ireland; 10 Physikalisch-Technische Bundesanstalt (PTB), Berlin, Germany; 11 NeuroSpin, CEA, Université Paris-Saclay, F-91191 Gif-sur-Yvette, France; 12 Institut National de la Santé et de la Recherche Médicale, UMR 992 INSERM, CEA, Faculté de médecine, Université Paris-Sud, Université Paris-Saclay, NeuroSpin, F-91191 Gif-sur-Yvette, France; 13 Institute of Medical Psychology and Medical Sociology, University Medical Center Schleswig-Holstein, Kiel University, Kiel, Germany; 14 Klinik für Kinder- und Jugendpsychiatrie- und Psychotherapie, Universitätsklinikum Heidelberg, Heidelberg, Germany; 15 Department of Psychological Medicine, Section for Eating Disorders, Institute of Psychiatry, Psychology and Neuroscience, King’s College London, London, SE5 8AF UK; 16 South London and Maudsley NHS Foundation Trust, London, UK; 17 Clinical and Experimental Sciences, Faculty of Medicine, University of Southampton, Southampton, UK; 18 Division of Psychiatry and Department of Clinical, Educational & Health Psychology, University College London, London, UK; 19 School of Psychology and Global Brain Health Institute, Trinity College Dublin, Dublin, Ireland; 20 Centre for Population Neuroscience and Stratified Medicine (PONS), Institute for Science and Technology of Brain Inspired Intelligence and National Center for Neurological Disorders, Huashan Hospital, Fudan University, Shanghai, China; 21 Department of Psychiatry, University of Cambridge, Cambridge, UK; 22 The German Center for Mental Health (DZPG), Charité Universitätsmedizin Berlin, Berlin, Germany

**Keywords:** Psychiatric disorders, Biomarkers

## Abstract

The STRATIFY (Brain Network-Based Stratification of Reinforcement-Related Disorders) and ESTRA (Eating Disorders Stratification) studies were established as harmonised “sibling” cohorts to develop a mechanistically informed framework for stratifying psychiatric disorders. Here, we describe the study design, methodology, and cohort characteristics. Both studies investigate how network properties of brain structure and function, together with biological markers derived from blood-based genomics, epigenetics, and proteomics, relate to reinforcement-related behaviours that cut across major depressive disorder, alcohol use disorder, psychosis, and eating disorders. A further objective is to identify discriminative multimodal features that predict disease onset, symptom course, and functional outcomes, thereby supporting the development of targeted interventions. STRATIFY and ESTRA recruited 674 patients and 70 healthy controls aged 18–30 years (76% females), supplemented by 199 age- and sex-matched healthy controls from the population-based IMAGEN cohort assessed at the same sites using harmonised protocols. Multimodal assessment included structured clinical interviews, self-report measures, cognitive testing, biosamples for molecular analyses, and multimodal MRI (structural, diffusion, resting-state, and task-based fMRI). ESTRA participants additionally completed longitudinal follow-up, and all cohorts were assessed during the COVID-19 pandemic. STRATIFY and ESTRA together constitute a large-scale, open-science resource integrating multimodal brain, behavioural, and biological data across transdiagnostic patient cohorts in early adulthood. The anonymised dataset is available to the research community through managed access, supporting international collaboration and accelerating the development of mechanistically informed classification systems and predictive tools in psychiatry.

## Introduction

Mental health disorders represent a significant public health concern, accounting for 14.6% of years lived with disability (YLD) [1] and contributing to 16% of the disease burden [2], measured as disability-adjusted life years (DALY), among non-communicable diseases. With their most pronounced impact occurring among adults of working age, the economic costs of mental health disorders are also substantial, reaching €600 billion in Europe in 2018 [3]. The societal burden of YLD due to mental-health conditions have surged by 48.1% since 1990, with the greatest increases observed for eating disorders (+15%), and for drug use (+17%) [4]. In terms of YLD, depressive disorders are identified as the leading contributor, accounting for 37.3% of global YLD, with psychosis at 12.2% in 2019 [1].

Critically, these diagnoses often co-occur, reflecting high levels of comorbidity [5–8]. For example, reinforcement-related conditions, such as depression, addiction and psychosis, exhibit strong comorbid relationships, where diagnosis of one mental health disorder elevates the risk for all other mental health conditions [9, 10]. This comorbidity could stem from aberrations in shared neurobehavioral reinforcement processes, including dysregulation within fronto-limbic-striatal [11–13] and fronto-hippocampal [14] circuits. Additionally, these processes maybe influenced by chemical modulatory systems such as monoaminergic, opioid, and glutamate neurotransmission [15, 16]. Genetic pleiotropy further indicates co-heritability, whereby specific groups of genes influence neural processes in a transdiagnostic manner, conferring risk for more than one disorder [17–20].

Beyond comorbidity, mental health disorders are marked by substantial within-diagnosis heterogeneity, where individuals with the same diagnostic label may differ in symptom profiles, underlying mechanisms, and treatment responses [21, 22]. Conversely, distinct disorders often share overlapping circuit dysfunction. Because current diagnostic systems are based on clinical presentation rather than biology, reliability remains imperfect and biological specificity limited [23, 24]. These challenges have hindered efforts to identify robust biomarkers and predictive tools. Contemporary approaches such as computational psychiatry and the RDoC framework emphasise the study of core cognitive-affective processes, including reinforcement learning, valuation, salience processing, and behavioural control, that may cut across traditional categories and provide a more mechanistic basis for stratification [25–28]. A stratification framework grounded in biological mechanisms holds potential to unravel both shared (comorbid) and unique (heterogeneous) features of mental health disorders, advancing our understanding of their aetiology and informing targeted interventions.

Importantly, most mental health conditions reach peak incidence during the second and third decades of life [29], with 75% developing by the mid-20s [30]. Epidemiological evidence consistently indicates that nearly three-quarters of clinically significant mental health disorders originate before the age of 25 [31, 32]. Moreover, earlier onset predicts prolonged symptom duration, increased comorbidities, and poorer outcomes [33], highlighting the need for investigation during this period.

Within this context, the STRATIFY (Brain Network-Based Stratification of Reinforcement-Related Disorders) and ESTRA (Eating Disorders Stratification) studies were initiated as harmonised “sibling” cohorts sharing common infrastructure and assessment protocols. This protocol paper introduces these two linked studies, designed to facilitate transdiagnostic investigation into the neurobiology of mental health disorders. The anonymised dataset is now available, supporting international collaboration and accelerating development of mechanistically informed stratification markers and therapeutic targets.

## Research protocol

The STRATIFY and ESTRA studies aims to advance a biologically grounded classification of psychiatric disorders. Anchored in quantifiable neurobehavioral constructs the studies target four major diagnostic groups: Major Depressive Disorder (MDD), Alcohol Use Disorder (AUD), Psychosis, and Eating Disorders (EDs), including Anorexia Nervosa (AN), Bulimia Nervosa (BN), and Binge Eating Disorder (BED).

### Study rationale

STRATIFY and ESTRA were deliberately designed as “sibling” studies to investigate reinforcement-learning and affect-valuation processes as transdiagnostic mechanisms in psychopathology. Converging behavioural, neuroimaging, and computational-modelling evidence indicates that alterations in reward learning, inhibitory control, salience attribution and value-based decision-making contribute to symptom formation in MDD, AUD, psychosis, and EDs [34–39]. These processes map onto overlapping fronto-striatal, limbic, and default-mode networks and are linked to dopaminergic prediction-error signalling [35–37]. Harmonised experimental paradigms therefore enable hypothesis-driven, mechanistically informed comparisons across clinically distinct groups, allowing investigation of both shared-transdiagnostic and disorder-specific circuity.

Within this framework, STRATIFY focused on MDD, AUD, and psychosis because these conditions are highly prevalent and clinically burdensome in young adulthood [1, 4]; exhibit substantial comorbidity [5–8], enabling transdiagnostic inference and show strong mechanistic links to reinforcement-learning and inhibitory-control circuitry [11–14]. AUD was prioritised over other substance-use disorders because validated phenotyping and task paradigms were harmonised with the IMAGEN cohort [40, 41], facilitating cross-cohort validation and statistical integration. Further, unlike illicit drug use, alcohol exposure is relatively homogeneous across Europe, facilitating recruitment. Although patients with other substance-use disorders were not recruited as primary diagnostic groups, detailed assessment of nicotine, cannabis, and other drug use was obtained for all participants, allowing these exposures to be modelled dimensionally in secondary analyses.

ESTRA extends this framework to EDs, in which reinforcement-learning, habit formation, threat-avoidance, and interoceptive valuation have also been implicated [37–39]. All conditions contribute substantially to global disease burden and were supported by existing specialist clinical services and recruitment pathways across participating sites. Together, these studies provide a coordinated platform for developing biologically grounded, mechanism-based stratification models. A harmonised multimodal battery spanning psychology, cognition, neurocognitive tasks, neuroimaging, and biospecimens were employed. These data allow estimation of reinforcement-learning parameters, brain-network organisation, and molecular signatures. Stratification is therefore anchored in mechanistic constructs rather than diagnostic labels, enabling identification of neurobehavioral subtypes across and within disorders.

### Study design

**Fig. 1:**
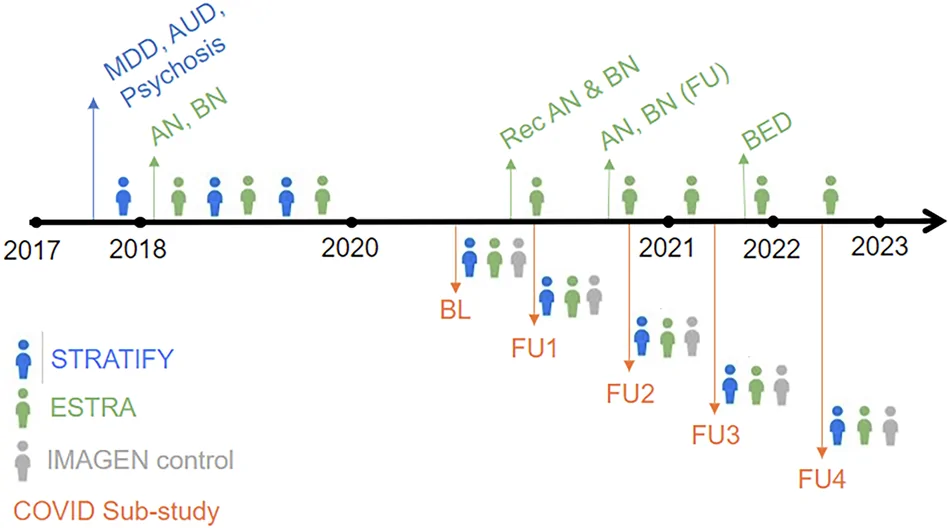
Study timeline. MDD: major depressive disorder, AUD: alcohol use disorder, AN: Anorexia nervosa, BN: Bulimia nervosa, BL: Baseline, FU: Follow-Up, Rec: Recovered, BED: Binge Eating Disorder.

The overarching aims of STRATIFY and ESTRA were to investigate if network connectivity (i) align with conventional diagnostic categories, (ii) correlate with symptom clusters within diagnoses, and (iii) span across diagnoses to elucidate patterns of comorbidity. Additionally, the studies sought to (iv) determine which data modalities best discriminate between identified groups and (v) best predict future psychopathology. Recruitment began in June 2017, with baseline assessments halted during the COVID-19 pandemic. During this time protocols were adapted to incorporate remote assessments, enabling pandemic exposure to be modelled explicitly as a covariate or moderator rather than treated as an uncontrolled confound. Follow-up evaluations, conducted during and after the lockdowns (Fig. 1), captured changes in mental, behavioural, and physical health, allowing longitudinal characterisation of resilience, vulnerability, and stress-related symptom dynamics.

The ESTRA follow-up study was conducted approximately 2.5 years after baseline in participants with AN or BN and HC, and was enriched with individuals recovered from AN or BN to identify neurobiological and psychological markers of illness course and remission. Together, our design (pre-COVID MRI → COVID-period phenotyping → post-COVID MRI) enables tests of whether pre-existing brain network organisation predicts vulnerability to pandemic-related psychopathology, whether pandemic exposure induces brain changes beyond normative developmental trajectories, and whether these effects differ between clinical and population samples.

All procedures received ethical approval from the London Westminster Research Ethics Committee and the Charité Ethikkommission (STRATIFY), and the Northwest–Greater Manchester South Research Ethics Committee (ESTRA). Written informed consent was obtained from all participants. Funding was provided by the European Research Council, UK Medical Research Council, and Medical Research Foundation.

### Inclusion and exclusion criteria

Participants were recruited from three European research sites: King’s College London (UK), University of Southampton (UK), and Charité Universitätsmedizin Berlin (Germany). Recruitment occurred through outpatient and inpatient services at affiliated hospitals: South London and Maudsley NHS Foundation Trust (SLaM), King’s College Hospital, University Hospital Southampton, and Charité Hospital Berlin; as well as via flyers and targeted online advertising. General exclusion criteria applied across all groups were: history of significant brain injury (e.g., stroke, epilepsy, tumour), neurodegenerative or other major neurological conditions, deafness or non-removable hearing aids, uncorrected visual impairment (corrected near vision ≤ 20/100 in both eyes), pregnancy or suspected pregnancy, type I or II diabetes, or use of medications for serious medical conditions unrelated to mental health. Individuals with restricted mobility preventing them from lying supine for 90 min were also excluded. Included participants were aged 18–30 years, of either sex. The 18–30 age range captures the modal onset period for the selected disorders [31, 32], maximising sensitivity to the mechanisms active during illness emergence and early course. Reinforcement-learning, salience-attribution, and inhibitory-control circuits, particularly within fronto-striatal and salience networks, continue to mature into the mid-twenties [42, 43]. Focusing on early adulthood therefore provides a developmentally stable period in which reinforcement-related circuitry is largely consolidated while remaining clinically informative. Inclusion criteria across participant group are as follows:

#### STRATIFY

Participants meeting diagnostic criteria for (a) unipolar major depression: based on a total score of ≥ 15 on Patient Health Questionnaire (PHQ-9) [44], (b) alcohol use disorder: based on a total score of ≥ 15 on Alcohol Use Disorders Identification Test (AUDIT) [45], (c) psychosis: based on F20-F29 ICD-10 [46] diagnosis by a psychiatrist, and healthy controls (HCs): PHQ-9 and AUDIT scores <5; no personal or familial history of psychiatric illness; no regular medication use; no learning disabilities; and no regular recreational drug use.

#### ESTRA, baseline study

Individuals meeting DSM-5 based diagnostic criteria for AN, BN, and BED, as assessed with the Eating Disorder Diagnostic Scale (EDDS) – DSM-5 version [47]. For current AN, a BMI < 18.5 kg/m² was required at baseline. Participants were recruited as ESTRA HCs if they reported no ED behaviours (binge, purge, exercising, fasting, or other) for the past 3 months, answered “not at all” or “slightly” to the first 3 questions of EDDS about feeling fat, fear of weight gain/becoming fat, and the influence of weight and shape on self-evaluation, and also met the inclusion criteria for STRATIFY HCs.

#### ESTRA, follow-up study

Approximately 2.5 years after baseline, participants with AN, BN, and HCs were re-assessed using the full baseline battery to track psychological and biological changes over time. In addition, the follow-up sample was enriched with individuals recovered from AN and BN, identified using the Structured Clinical Interview for DSM-5, Research Version (SCID-5-RV) [48]. The original SCID-5-RV assesses recovery for the past 3 months, which was amended to assess recovery for the past 6 months. The 6-month recovery criterion was defined to distinguish stable recovery from transient symptom fluctuation and to ensure that neurobiological and behavioural markers are not driven by acute illness effects, thereby improving interpretability of comparisons between current, recovered and control groups. This duration captures clinically meaningful recovery while remaining sensitive to early remission trajectories [49]. Recovered AN participants had a history of AN based on the DSM-5 criteria and maintained recovery for ≥6 months. Recovered BN participants had a history of BN and maintained full or partial recovery for ≥6 months. Partial recovery was defined as binge/purge behaviours ≤2 times per month over the past 6 months, with residual cognitive symptoms, i.e., their self-evaluation might still be unduly influenced by body shape and weight.

#### IMAGEN as healthy controls

STRATIFY and ESTRA were designed as patient-focused stratification studies, with recruitment weighted toward clinical groups to optimise power for identifying neurobehavioral subtypes within and across diagnoses. To maintain a robust normative reference, a smaller contemporaneous control group was recruited and supplemented with healthy participants from the population-based IMAGEN cohort, collected at the same sites using harmonised protocols. IMAGEN is a large-scale European cohort that investigates the genetic, neurobiological, and environmental factors influencing mental health and brain development through longitudinal follow ups [50]. IMAGEN participants were screened free of psychiatric diagnoses across all assessment waves using the Development and Well-Being Assessment (DAWBA) and, at age 23, the Mini International Neuropsychiatric Interview (MINI). Age- and sex-matched participants from the Berlin and London sites were included to support normative comparisons. Matching was implemented across diagnosis using exact ratio constraints on sex and minimisation of group-wise absolute mean age difference.

## Assessments

To ensure the reproducibility of biomarkers for classifying and predicting network properties, and to evaluate their distribution, protocols were harmonised with the population-based IMAGEN study. For normative inference, equivalence of data-generating processes (protocols, scanners, tasks, and pipelines) is more critical than identical sampling frames [51]. By harmonising methodology with IMAGEN, STRATIFY/ESTRA enable valid cross-cohort comparison despite modest age-window differences. Further, all cross-cohort analyses will statistically adjust for age and sex and, where appropriate, restrict inferences to matched subsamples to minimise developmental confounding. A key strength of this design is the ability to apply normative-modelling frameworks [52, 53], in which each patient’s neuroimaging and behavioural profile is expressed as a deviation from age- and sex-adjusted population reference distributions. Normative modelling offers greater statistical efficiency than conventional case–control contrasts, enabling inference at the individual-participant level, and is well-suited to detecting transdiagnostic biomarkers.

Participants completed a standardised, multi-modal assessment protocol comprising both remote and in-person components. Remote assessments were administered via the Delosis platform (https://www.delosis.com/psytools/overview.html) and included validated self-report measures capturing personality traits, environmental exposures, substance use, psychopathology, and subjective well-being (see Table 1 for full list). In-person assessments consisted of: (a) standardised clinical interviews and computer-based cognitive tasks, (b) anthropometric measures (height and weight), (c) venous blood and urine collection, and (d) Magnetic Resonance Imaging (MRI) scans.

**Table 1:** List of assessments in STRATIFY/ESTRA.

Substance use	
Alcohol Use Disorders Identification Test (AUDIT)	Substance use is assessed using the Alcohol Use Disorders Identification Test (AUDIT [45]), which was developed and validated by the World Health Organization to assist the brief assessment of alcohol use disorders and was specifically designed for international use. It exists in multiple languages, including German, French and English, and was validated on primary health care patients in six countries.
European School Survey Project on Alcohol and Drugs (ESPAD)	For drugs other than alcohol, sections of the “European School Survey Project on Alcohol and Other Drugs” (ESPAD [84]) are used to obtain measures of age of onset and quantity and frequency of alcohol, and illicit drug use in one’s lifetime, past 12 months, past 30 days, and past week.
Fagerstrom Test for Nicotine Dependence (FTND)	The Fagerstrom Test for Nicotine Dependence (FTND [85]) is used to assess nicotine dependence and smoking frequency in the past 30 days.
Drinking Motives Questionnaire (DMQ)	The Drinking Motives Questionnaire - Revised (DMQ-R [86]) breaks the motivation to use alcohol into 4 components: Drinking to cope with negative affect, Drinking to enhance positive affect, Drinking to be sociable and Drinking to conform to a group.
Rutgers Alcohol Problem Index (RAPI)	An 18-item self-administered screening tool for assessing adolescent problem drinking [87]. It was developed in order to create a conceptually sound, unidimensional, relatively brief, and easily administered instrument to assess problem drinking in adolescence. The advantages of this screening tool lie in its ease of administration and its standardization, which make it possible to compare problem drinking scores not only across individuals but also across groups. The index is to be used as a continuous variable which indicates the frequency of experiencing negative consequences due to alcohol use.
Drug Abuse Screening Test (DAST)	The DAST is one of the most widely used screening tests for drug abuse and addiction [88].
SCID – Substance Use Disorder (SCID - SUD)	The SCID is a semi-structured diagnostic instrument that provides lifetime diagnoses (past and current) for substance use disorders following DSM-IV nosology. In clinical research studies, the SCID has demonstrated good reliability and vali [89].
Michigan Alcoholism Screening Test (MAST)	The MAST screening tool is a 25-question test that is used to help identify an alcohol dependency [90].
Timeline-Followback Interview (TLFB)	A Timeline-Followback Interview [91] is conducted on the day of neuroimaging for the last 30 days on use of alcohol, tobacco and various drugs to further assess the reliability of self-report. The TLFB is a method of assessing substance use using memory prompts to optimise on the reliability and accuracy of self-report. It provides rich information on current substance use patterns.
**Temperament**	
NEO Five-Factor Inventory (NEO-FFI)	The NEO-FFI is a valid method of assessing broad dimensions of personality based on the Five-Factor Model of personality [92].
Temperament and Character Inventory (TCI)	The novelty seeking scale of the Temperament and Character Inventory – Revised (TCI-R [93]), was included to assess lower order trait dimensions more specifically related to disinhibitory psychopathology.
Substance Use Risk Profile Scale (SURPS)	The Substance Use Risk Profile Scale (SURPS [94]) assesses levels of several lower order personality traits for substance abuse/dependence and psychopathology including hopelessness, anxiety sensitivity, impulsivity and sensation seeking.
Barratt Impulsivity Scale (BIS-11)	The Barratt Impulsivity Scale (BIS [95]) is a 30-item self-report questionnaire. The BIS has three subscales labelled attentional, non-planning and motor (Stanford et al., 2009). Participants are asked to rate how often a particular statement would apply to them using a Likert scale ranging from 1 (rarely/never) to 4 (almost always/always), an example item is “I plan tasks carefully”. Since no timeframe is specified it is considered to be a measure of trait impulsivity. A total response score of 72 or higher classifies an individual as highly impulsive.
**Cognition**	
Monetary-Choice Questionnaire (KIRBY)	The Monetary-Choice Questionnaire (KIRBY [96]) provides a measure of preference of immediate lower over delayed higher monetary rewards. This measure asks for relative preference of one sum compared to another sum rather than asking for decisions about absolute amounts of money.
Stimulus Response compatibility (SRC)	The SRC task was introduced by De Houwer et al. [97]. It aims to measure the affective Simon effect. This effect occurs when someone is inclined to respond in a certain way to a stimulus, i.e. faster for congruent than for incongruent responses. For example: someone that would like to drink alcohol would be more inclined to respond to an alcohol stimulus by approaching it (congruent, faster response), than by avoiding it (incongruent, slower response).
Passive Avoidance Learning Paradigm (PALP)	This Go/No Go task is an experimental method for investigating passive avoidance learning and behavioral disinhibition [98]. Passive avoidance is defined as the ability to withhold a response that would have led to punishment. In this task subjects must learn by trial and error to response to “good” numbers for monetary reward and withhold response to “bad” numbers to avoid punishment (loss of money).
**Life-events**	
Life Events Questionnaire (LEQ)	The Life-Events Questionnaire (LEQ) is an adaptation of the Stressful Life-Event Questionnaire from Newcomb et al. [99], that was originally validated on a US sample, and was adapted to the demands of our UK/German and French sample in terms of wording. The scale uses 39 items to measure the occurrence (“ever”, “in the past year”) and the perceived desirability of events covering the following domains: Family/Parents, Accident/lllness, Sexuality, Autonomy, Deviance, Relocation, and Distress.
Bully Questionnaire (BULLY)	The bullying questions have been adapted from a questionnaire used in a large international study entitled Health Behaviour in School-aged Children (HBSC). These questions were initially utilised in the revised Olweus Bully/Victim Questionnaire [100], and the Youth Risky Behaviour Survey [101].
**Mental Health**	
Center for Epidemiologic Studies Depression Scale (CES-D)	Center for Epidemiological Studies Depression Scale (CES-D [102]) is a short (20-item) self-report scaled used to measure depression symptoms and has been used extensively in research.
The Three-Factor Eating Questionnaire (TFEQ)	The TFEQ is a short version (18 items) of one of the most widely used measures in the field of eating behaviour research. It measures cognitive restraint, uncontrolled eating and emotional eating. It has good psychometric properties and has been used in different European populations [103, 104].
Eating Disorder Diagnostic Scale-DSM-IV version (EDDS DSM-IV)	The EDDS (DSM-IV version) is a 22-item self-report questionnaire designed to assess eating disorder diagnoses and generate an overall composite score of eating disorder symptoms [105].
Eating Disorder Examination Questionnaire (EDEQ)	The EDEQ is a widely used 28-item self-report questionnaire [106]. Reliability and validity of the measure are well established. It measures eating disorder behaviours (e.g. bingeing, vomiting, purging) and attitudinal aspects (Restraint, Eating Concern, Weight Concern and Shape Concern) over the previous 28 days.
Community Assessment of Psychic Experience (CAPE-42)	CAPE Community Assessment of Psychic Experience – CAPE-42 [107]. Various terms have been used to refer to the sub-clinical manifestations of the psychosis phenotype, including psychosis proneness, psychotic-like experiences (PLEs) and schizotypy.
Brief Symptom Inventory (BSI-53)	The BSI [108] is the shortened version of the Symptoms Checklist-90. The BSI is a 53-item questionnaire covering nine psychological symptoms: 1) Somatisation, 2) Obsession-compulsion, 3) Interpersonal sensitivity, 4) Depression, 5) Anxiety, 6) Hostility, 7) Phobic anxiety, 8) Paranoid ideation, and 9) Psychoticism. The BSI is one of the most widely accepted psychometric screening tools of general psychopathology in both research and clinical practice.
Patient Health Questionnaire - 9 (PHQ-9)	The Patient Health Questionnaire (PHQ [109]) is a self-administered tool used to screen and assess the severity of depression, anxiety, and other mental health disorders. The PHQ is widely used because of its simplicity and reliability in identifying potential mental health issues. The last question in PHQ-9 (originally asking about suicidality and self-harm) was adapted as “How DIFFICULT have these problems made it to do your work, take care of things at home, or get along with other people?”.
Anxiety Screening from the Composite International Diagnostic Interview (CIDI)	The screening process in the CIDI [110] involves asking individuals about specific anxiety symptoms they may have experienced over a defined period, such as the past month or year. Questions cover various domains of anxiety, including worry, nervousness, avoidance behaviors, panic attacks, and specific fears or phobias.
Children Somatization Inventory (CSI)	A shortened version of the Children’s Somatization Inventory Revised Form (CSI-24 [111]) is suitable for use in adults (AL Sherman 2012). The CSI includes items from the symptom criteria for somatization disorder as defined by the DSM-III-R (American Psychiatric Association, 1987), items from the Somatization factor of the Hopkins Symptom Checklist (HSCL [112]). The standard time period for symptom report on the CSI is 2 weeks. The response format is a 5-point scale ranging from “not at all” (0) to “a whole lot”(4).
Strengths and Difficulties Questionnaire (SDQ)	The self-report Strengths and Difficulties Questionnaire (SDQ [113]) was used to assess 5 dimensions of youth pro-social and antisocial behaviour: emotional symptoms, conduct problems, hyperactivity/ inattention, peer relationship problems, and prosocial behaviour. The SDQ is a reliable and valid measure of youth emotional and behaviour symptoms, on which extreme scores are predictive of increased probability of clinician-rated psychiatric disorders and retest stability over 4-6 months [114].
The Development and Well-Being Assessment Interview (DAWBA)	The Development and Well-Being Assessment (DAWBA [115]) is a computer-based package of questionnaires, interviews, and rating techniques designed to generate ICD-10 and DSM-IV psychiatric diagnoses. The different types of information is brought together by a computer program which also predicts the likelihood of diagnosis. These computer-generated summary sheets and diagnoses are reviewed by experienced clinical raters, who decide to either accept or overturn the computer diagnosis (or lack of diagnosis).
Mini-International Neuropsychiatric Interview Version 5 (MINI-5)	A face-to-face psychopathological assessment using the Mini-International Neuropsychiatric Interview version 5.0 (MINI [116]) is administered during the Institute assessment. The MINI is a structured diagnostic interview designed to assess the presence of current DSM-IV and ICD-10 psychiatric disorders, which will be evaluated for its relationship with neuroimaging, substance use and psychiatric comorbidity. The sub-diagnoses covered in the MINI are Major Depressive Episode, Dysthymia, Suicidality, Manic/Hypomanic Episode, Panic Disorder, Agoraphobia, Social Phobia, Obsessive-Compulsive Disorder, Alcohol Dependence/Abuse, Substance Dependence/Abuse, Psychotic Disorders, Anorexia Nervosa and Bulimia Nervosa.
**General**	
Early and Current Urbanicity	A short questionnaire assessing current and early urbanicity assesses the link between urbanicity, social stress sensitivity and mental disorders. Participants are asked about where they lived up until age 15 in terms of population size in order to determine the urbanicity of their upbringing. Several articles have shown an association between urbanicity and symptoms of psychosis, depression and anxiety. Urban upbringing and city living have impacts on social evaluative stress processing in humans, resulting in specific effects on regional brain activation [117].
Video-Gaming Scale	This assesses video gaming addiction [118]. The questionnaire contains 26 items.
Mobile phone usage questionnaire (SCAMP)	A 13-item questionnaire modified from the SCAMP study [119]. It asks about the mobile phone usage (i.e., frequency of phone calls, texting/messaging behavior, internet/App usage).
**Well-being**	
World Health Organization Well-Being Index (WHO-5)	The WHO-5 Well-Being Index is a 5-item questionnaire that measures current emotional well-being. It is a short, self-administered questionnaire covering 5 positively worded items, related to positive mood (good spirits, relaxation), vitality (being active and waking up fresh and rested), and general interests (being interested in things). It has shown to be a reliable measure of emotional functioning and a good screener for depression (www.dawnstudy.com) [120].
Positive and Negative Affective Scale (PANAS)	The Positive and Negative Affect Schedule (PANAS [121]) is a 20-item self-report measure of positive and negative affect developed by Watson, Clark, and Tellegen (1988b). It asks participants to rate the extent to which they experienced each feeling/emotion on a 5-point Likert Scale ranging from “very slightly” to “very much”. The PANAS is believed to provide independent measures of Positive Affect (PA) and Negative Affect (NA). Since its development the measure has been employed in research for diverse purposes. Its popularity is attributed to its brevity and its close association with an influential conceptualization of anxiety and depression: the tripartite model.
Kessler-6 Psychological Distress Scale (K6+)	The six questions in the K6+ [122] cover various aspects of psychological distress, including feelings of nervousness, hopelessness, restlessness, and depressive symptoms. Respondents rate each item on a 5-point Likert scale based on how frequently they experienced each symptom, ranging from “None of the time” to “All of the time.”
Perceived Stress Scale - I0 items (PSS-10)	The Perceived Stress Scale (PSS [123]) is a widely used self-report instrument to measure nonspecific stress (S Cohen et al. 1983). The original version contained 14 items but a shorter version will be used this study. It consists of 10 questions asking how often in the last month has their life been difficult/stressful. The item responses are on a 5-point Likert scale ranging from “Never” to “Very Often”. Measuring non-specific stress, compared to only measuring specific stressors, is important as it accounts for the subjective interpretation of stressors. This subjective interpretation may influence how a person experiences and manages a particular stressor.
Health-related quality of life (HRQOL)	Health-related quality of life (HRQOL [124]) is a multidimensional concept that encompasses an individual’s physical, mental, emotional, and social well-being in relation to health and healthcare.
Health/Function questions from CDC Behavioural Risk Factor Surveillance System Survey (BRFSS)	The Centers for Disease Control and Prevention (CDC) Behavioral Risk Factor Surveillance System (BRFSS [125]) is a survey used to ask US residents about health risks and chronic conditions (http://www.cdc.gov/brfss/questionnaires/index.htm). Psychopathology, such as depression, often presents itself with somatic comorbidities (i.e., cardiovascular disease, cancer, diabetes). Therefore, 18 questions have been selected asking about their general physical health, if their health has affected their usual activities, sleep frequency/quality, and whether or not they’ve been diagnosed with chronic conditions (i.e., heart problems, stroke, cancer, kidney disease, diabetes).
Interpersonal Reactivity Index (IRI)	The IRI is a well-validated 28-item self-report questionnaire of dispositional empathy comprising four separate but related constructs. Four seven-item subscales tap 1) perspective taking (PT), the tendency to spontaneously adopt the psychological point of view of others in everyday life; 2) empathic concern (EC), the tendency to experience feelings of sympathy and compassion for unfortunate others, 3) personal distress (PD), the tendency to experience distress and discomfort in response to extreme distress in others, and 4) fantasy (FS), the tendency to imaginatively transpose oneself into fictional situations. The IRI has been well-validated and is in itself routinely used as a validation tool routinely [126] and widely used in both community and clinical samples.

### Biological samples and multi-omics data acquisition

Venous blood (2 × 10ml EDTA tubes and 2 × 3ml tempus tubes) was collected by trained nurses. Within an hour of collection, one EDTA tube was centrifuged at 4 °C for 10 min at 2000g and aliquoted to separate plasma, buffy coat and red blood cells samples. These aliquots along with the tempus tubes (for RNA isolation) and second EDTA (for DNA extraction) were stored at −80 °C. Urine samples (2 × 10ml monovette tubes) were also collected and stored at −80 °C. Genetic and multi-omics data have been acquired for the STRATIFY, ESTRA-AN, and ESTRA-BN samples. These data will be used for exploratory biomarker analyses and will allow ancestry-related structure to be derived analytically and modelled directly in future analyses, for example through the inclusion of genetic principal components. The following multi-omics data have been acquired and are available on request: genome-wide genotyping (Infinium Global Screening Array), DNA methylation (Infinium Methylation EPIC Bead Chip), RNA sequencing (Lexogen QuantSeq 3’mRNA-Seq) and high-throughput proteomics (Scanning SWATH). Remaining aliquots are retained under controlled access for future analyses in line with participants’ consent.

### Neuroimaging data

MRI data were acquired on 3T scanners at each site using harmonised protocols (across sites and cohorts). Southampton used Siemens Magnetom Skyra with multi-channel head coil; Berlin used Siemens Magnetom Verio with multi-channel head coil; London used 2 scanners: Siemens Magnetom TrioTim with 32 channel head coil and GE Healthcare MR750 system with 8-channel high-resolution head coil. Sequence timing, voxel resolution, and acquisition protocols remained stable throughout data collection (details in Table 2); for functional MRI (fMRI), parameters were directly matched between sites while structural parameters were harmonized at the level of final image appearance, with some parameters differing to accommodate manufacturer-specific implementations. Standard quality-assurance procedures were used across sites, including phantom-based calibration and scanner performance monitoring; nonetheless researchers are recommended to use scanner site as covariate in all analysis to account for residual inter-scanner variability. Structural anatomical scans were acquired using T1-weighted 3D MPRAGE; T2-weighted turbo/fast spin echo and FLAIR imaging. fMRI data were collected using gradient-echo EPI sequences. Whole-brain fMRI coverage was acquired with a slice thickness of 2.4 mm and a 1 mm inter-slice gap, using a descending (superior-to-inferior) slice order. In-plane acquisition used a 64×64 matrix over a 21.8×21.8 cm field of view, yielding an in-plane voxel size of 3.4 mm. Parallel imaging acceleration (GRAPPA/ASSET factor = 2) and fat suppression were applied. Task-based fMRI included Monetary Incentive Delay task (MID [54]), Emotional Face-processing task (EFT [55]), and Stop-Signal task (SST [56]), with 191, 202, and 349 volumes respectively. Resting-state fMRI comprised 164 volumes acquired with identical parameters. Diffusion tensor imaging (DTI) data were acquired using single-shot spin-echo EPI with bipolar diffusion gradients. Diffusion weighting included 36 non-collinear directions at b = 1300 s/mm² and at least one b = 0 image with parallel imaging (acceleration factor 2). Further, to evaluate white matter connectivity, neurite orientation dispersion and density imaging (NODDI) scans were acquired using multiband single-shot spin-echo EPI sequence. Diffusion weighting included a high b-value shell (b = 2000s/mm^2^) with 128 diffusion directions, a low b-value shell (b = 700 s/mm^2^) with 64 diffusion directions together with 8 non-diffusion-weighted images. Four volumes were acquired with b = 0 and a reversed EPI-phase encoding gradient. Multiband acceleration factor of 3 and parallel imaging were used to reduce acquisition time and susceptibility related distortions. To correct for susceptibility-induced geometric distortions, dual-echo gradient-echo field maps were acquired. Pre-scan normalization, and visual inspection of raw images were completed at each site.

**Table 2:** MRI acquisition parameters.

	Slices	FoV	Slice thickness	TR	TE	FA	TI/Acceleration technique
**T1 ADNI MPRAGE**							
Berlin	160	280 mm	1.1 mm	2300 ms	2.93 ms	9	900 ms
Southampton	176	256 mm	1.0 mm	2300 ms	2.98 ms	9	900 ms
London Siemens	160	280 mm	1.1 mm	2300 ms	2.93 ms	9	900 ms
London GE	160	270 mm	1.2 mm	7.328 ms	3.02 ms	11	400 ms
**T2**							
Berlin	36	240 mm	4.0 mm	4380 ms	61 ms		GRAPPA
Southampton	36	240 mm	4.0 mm	4380 ms	63 ms		GRAPPA
London Siemens	36	240 mm	4.0 mm	4380 ms	60 ms		GRAPPA
London GE	36	240 mm	4.0 mm	4380 ms	60 ms		ASSET
**FLAIR**							
Berlin	36	240 mm	4.0 mm	8000 ms	119 ms		2000 ms
Southampton	36	220 mm	4.0 mm	9000 ms	81 ms		2500 ms
London Siemens	36	240 mm	4.0 mm	8000 ms	119 ms		2000 ms
London GE	36	240 mm	4.0 mm	8000 ms	120 ms		2000 ms
**fMRI**							
Berlin	40	220 mm	2.4 mm	2200 ms	30 ms	75	GRAPPA
Southampton	40	218 mm	2.4 mm	2200 ms	30 ms	75	GRAPPA
London Simens	40	220 mm	2.4 mm	2200 ms	30 ms	75	GRAPPA
London GE	40	218 mm	2.4 mm	2200 ms	30 ms	75	ASSET
**DTI**							
Berlin	60	307 mm	2.4 mm	15000 ms	104 ms		GRAPPA
Southampton	60	307 mm	2.4 mm	15000 ms	104 ms		GRAPPA
London Simens	60	363 mm	2.4 mm	15000 ms	117 ms		GRAPPA
London GE	60	307 mm	2.4 mm	18462 ms	85 ms		ASSET
**NODDI**							
Berlin	66	256 mm	2.0 mm	3500 ms	99 ms		Multiband
London Siemens	66	256 mm	2.0 mm	3500 ms	100 ms		Multiband
London GE	66	256 mm	2.0 mm	3500 ms	97 ms		Multiband

Note that for MPRAGE scans, the definition of TR, and this its value, differs between manufacturers.

*FoV*, Field of View; *TR*, repetition time; *TE*, echo time; *FA*, flip angle.

During the COVID-19 pandemic, protocols were adapted to capture the impact of lockdowns and social distancing measures. Additional self-report measures assessed changes in mental health, lifestyle, substance use, social functioning, and media consumption. COVID-specific items were adapted from the Coronavirus Health Impact Survey (CRISIS) [57]. Parallel assessments were administered in the IMAGEN cohort, enabling comparative analyses of clinical and general population trajectories. A comprehensive list of assessments administered at each timepoint is provided in Table 3.

**Table 3:** Questionnaire administered during COVID follow up waves.

Time-point	Questionnaires administered
Baseline. April 2020. During the first lockdown.	CRISIS questionnaire, AUDIT, EDEQ, TFEQ, WHO-5, Kirby MCQ, PANAS, K6 + , CSI, BSI, PHQ-9
FU1. July 2020. After the first lockdown.	CRISIS questionnaire
FU2. November 2020. During the second lockdown.	CRISIS questionnaire, AUDIT, PANAS, BSI, PHQ-9, EDEQ
FU3. April 2021. After the second lockdown.	CRISIS questionnaire, AUDIT, PANAS, BSI, PHQ-9, EDEQ
FU4. June 2022. Post pandemic.	CRISIS questionnaire + full baseline battery + MRI scans

The last question in PHQ-9 (originally asking about suicidality and self-harm) was adapted as “How DIFFICULT have these problems made it to do your work, take care of things at home, or get along with other people?”.

*FU*, follow-up; *CRISIS*, the CoRonavIruS Health Impact Survey; *AUDIT*, Alcohol Use Disorders Identification Test; *EDE-Q*, Eating Disorder Examination Questionnaire; *TFEQ*, Three-Factor Eating Questionnaire; *WHO-5*, World Health Organisation- Five Well-Being Index; *MCQ*, Monetary Choice Questionnaire; *PANAS*, Positive and Negative Affect Schedule; *K6+*, Kessler Psychological Distress Questionnaire; *CSI*, Central Sensitisation Inventory; *BSI*, Brief Symptom Inventory; *PHQ-9*, Patient Health Questionnaire-9.

## Data collection

### STRATIFY and ESTRA baseline study

**Fig. 2:**
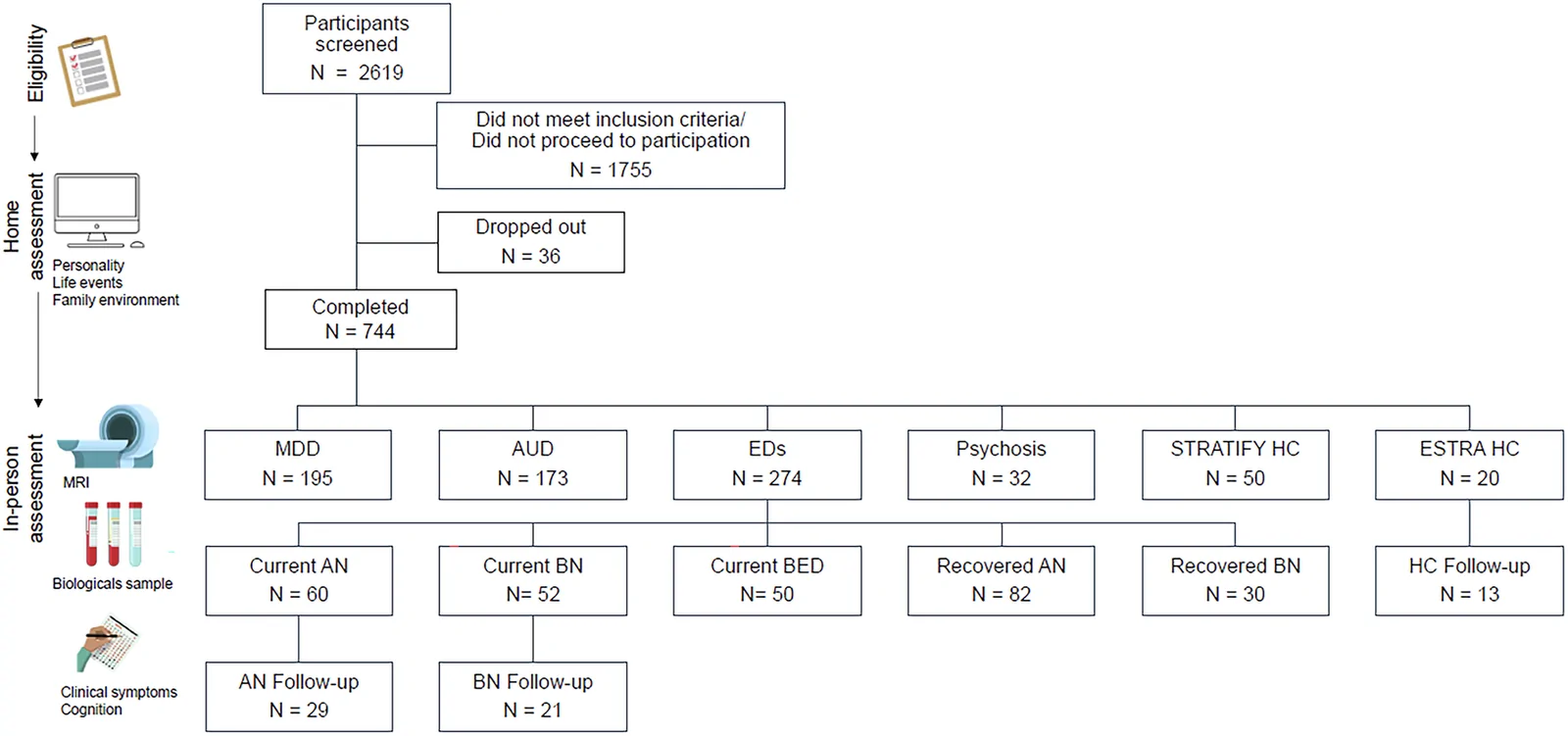
STROBE flow chart. MDD: major depressive disorder, AUD: alcohol use disorder, EDs: eating disorders, AN: anorexia nervosa; BN: bulimia nervosa; BED: binge eating disorder; HC: healthy controls.

Recruitment for the STRATIFY and ESTRA studies began in June 2017. As illustrated in the STROBE diagram (Fig. 2), 2619 individuals were screened, and 780 (~30%) met the inclusion criteria. Of these, 674 patients and 70 healthy controls (HCs) were successfully enrolled. This patient sample included 195 individuals diagnosed with MDD, 173 with AUD, 32 with psychosis, and 162 with EDs comprising 60 with current AN, 52 with current BN, and 50 with current BED. An additional 112 individuals who had recovered from EDs were also recruited (AN = 82; BN = 30). Notably, while female sex was not an inclusion criterion for AN/BN, only female participants in these categories volunteered for the study. Participants in the STRATIFY sample and the ESTRA anorexia nervosa and bulimia nervosa samples self-identified as being of White/European ancestry. Demographic characteristics including ethnicity information for all included participants are reported in Table 4. The control group included 50 STRATIFY HCs and 20 ESTRA HCs. An additional 199 HCs were selected from the IMAGEN study, to strengthen normative comparison.

**Table 4:** Summary of demographic information.

Group	N	Female N (%)	Age: Mean (SD)	Age range	White/European ethnicity (%)	Study site		
						London N (%)	Berlin N (%)	Southampton N (%)
MDD	195	142 (72.8%)	22.7 (2.2)	18.1–28.1	100%	100 (51.3%)	32 (16.4%)	63 (32.3%)
AUD	173	97 (56.1%)	23.0 (2.4)	18.3–30	100%	100 (57.8%)	21 (12.1%)	52 (30.1%)
Psychosis	32	12 (37.5%)	24.9 (3.3)	18–31.5	100%	10 (31.2%)	19 (59.4%)	3 (9.4%)
HC for STRATIFY	50	32 (64%)	22.5 (2)	18.3–25.6	100%	–	–	50 (100%)
HC from IMAGEN	199	102 (51.3%)	21.6 (1.5)	18.0–24.6	87.2%	113 (56.8%)	86 (43.2%)	–
AN	60	60 (100%)	21.8 (2.1)	18.2–26.2	100%	60 (100%)	–	–
BN	52	52 (100%)	22.0 (2.1)	18.8–28	100%	52 (100%)	–	–
BED	50	45 (90%)	23.2 (2.7)	18.4–29.4	58.1%	50 (100%)	–	–
Recovered AN	82	78 (95.1%)	23.2 (2.5)	19.1–29.3	79.7%	83 (100%)	–	–
Recovered BN	30	27 (90%)	23.5 (3)	18.2–30.1	75%	29 (100%)	–	–
HC for ESTRA	20	20 (100%)	23.4 (2)	19.8–25.9	100%	20 (100%)	–	–

age information was based on the date participants completed the online questionnaires on the Delosis platform.

### COVID follow-up study

Baseline assessments for the COVID-19 follow-up study were initiated during the national lockdown in April 2020, with 729 participants completing the baseline survey (458 IMAGEN; 271 STRATIFY/ESTRA). Confirmed SARS-CoV-2 infection was rare (2 STRATIFY/ESTRA and 9 IMAGEN participants). Consequently, COVID-19 effects in the present dataset predominantly index psychosocial stressors associated with the pandemic rather than the direct biological impact of infection. Sample sizes for subsequent waves are shown in Table 5. Dropout analyses showed no baseline differences in sex, depressive symptoms, ED symptoms, or harmful drinking between responders and non-responders (*p*s ≥ 0.1; Table 6). However, retention declined in several clinical groups at later waves (e.g., FU3: MDD 19%, AUD 12%, BN 11%), therefore attrition-related bias cannot be excluded, and longitudinal findings should be interpreted with caution.

**Table 5:** Number of participants in each phase of the COVID-19 assessments.

Group	BL	FU1	FU2	FU3
MDD	81	45 (56%)	38 (47%)	15 (19%)
AUD	51	36 (71%)	30 (59%)	6 (12%)
Psychosis	9	6 (67%)	6 (67%)	5 (56%)
AN	45	38 (84%)	31 (69%)	16 (36%)
BN	35	23 (66%)	20 (57%)	4 (11%)
Recovered AN	20	6 (30%)	6 (30%)	5 (25%)
Recovered BN	3	1 (33%)	0 (0%)	1 (33%)
STRATIFY HC	16	10 (63%)	9 (56%)	0 (0%)
ESTRA HC	11	8 (73%)	8 (73%)	1 (9%)
IMAGEN study	458	331 (72%)	317 (69%)	95 (21%)

Percentages in the parentheses are relative to the covid baseline assessment.

*BL*, baseline; *FU*, follow-up.

**Table 6:** Characteristics in COVID-19 baseline assessments of participants who attended the first follow-up (FU1) and those who did not attend.

	Measure	Dropped out from COVID FU1, % or Mean (SD)	Attended COVID FU1, % or Mean (SD)	*P*-value
Clinical sample	N	92	163	–
	Sex (female)	79.3%	86.5%	0.19
	ED symptom levels	2.1 (1.66)	2.5 (1.51)	0.072
	Depressive symptoms	11.56 (5.95)	12.56 (6.64)	0.28
	Harmful drinking	7.97 (8.64)	9.72 (9.21)	0.16
Population-based sample	N	127	331	–
	Sex (female)	56.7%	65.3%	0.11
	ED symptom levels	1.03 (1.03)	1.15 (1.14)	0.32
	Depressive symptoms	6.20 (5.23)	5.69 (4.29)	0.32
	Harmful drinking	6.12 (5.13)	5.47 (4.95)	0.24

All the patient groups in the clinical sample were pooled together, including MDD, AUD, Psychosis, AN, BN, recovered AN, and recovered BN.

*p*-values were obtained from chi-squared tests for the comparison of sex, and from independent samples t-tests for the comparison of the mental health symptoms. ED symptom levels were measured with EDE-Q total scores. Depressive symptoms were measured with PHQ-9 total scores. Harmful drinking was measured with AUDIT total scores.

### ESTRA follow-up study

A subset of ESTRA baseline participants with AN, BN, and ESTRA HCs were re-assessed approximately 2.55 years later (SD = 0.78). A total of 29 (48%) AN, 21 (34%) BN, and 13 (65%) ESTRA HCs participated in the follow-up. No significant baseline differences were found between AN participants who completed the follow-up and those lost to follow-up regarding ED symptoms, depression, or harmful alcohol use (Table 7). However, BN participants who returned for follow-up exhibited significantly higher ED symptoms at baseline than those who did not (*p* = 0.023, independent samples t-test).

**Table 7:** Baseline characteristics of participants who attended the follow-up assessment and those who did not attend.

Patient group	Measure	Attended the follow-up. Mean (SD)	Dropped out from the follow-up. Mean (SD)	*P*-value
AN	N	29	31	-
	ED symptom levels	0.28 (0.61)	0.26 (0.55)	0.917
	Depressive symptoms	15.07 (6.14)	12.94 (5.38)	0.157
	Harmful drinking	4.79 (3.96)	4.26 (4.43)	0.625
BN	N	21	31	-
	ED symptom levels	1.09 (0.26)	0.89 (0.32)	0.023
	Depressive symptoms	14.57 (6.38)	11.68 (6.25)	0.110
	Harmful drinking	6.43 (5.35)	8.10 (7.95)	0.405
ESTRA HC	N	13	7	-
	ED Symptom levels	−0.71 (0.18)	−0.82 (0.05)	0.159
	Depressive symptoms	3.38 (2.77)	2.80 (3.03)	0.732
	Harmful drinking	3.08 (1.71)	2.86 (1.57)	0.781

*P*-values were obtained from independent groups t-tests comparing those who dropped out with those who attended the follow-up. All the AN and BN participants were female and were recruited from the London study site. ED symptom levels were measured with EDDS composite scores. Depressive symptoms were measured with PHQ-9 total scores. Harmful drinking was measured with AUDIT total scores.

### MRI derived measures

All MRI data underwent harmonised preprocessing using validated pipelines aligned with IMAGEN and rigorous quality control, with pre-computed structural, functional, and diffusion-derived measures available to facilitate secondary analyses; minimally processed data are also available to enable derivation of additional metrics and ensure analytic reproducibility. T1-weighted scans were processed using FreeSurfer, including motion correction, skull-stripping, Talairach transformation, intensity normalisation, segmentation of cortical and subcortical structures, topology correction, and surface reconstruction [58]. Cortical thickness, grey-matter volumes, and surface-based morphometric measures were derived. Complementary grey-matter segmentations were generated using FSL and CAT12 (SPM**)**, including bias-field correction and spatial normalisation to MNI space to support voxel-based morphometry [59]. All anatomical images and derived segmentations were manually inspected for motion artefacts, structural abnormalities, and registration accuracy. Diffusion data were processed using MRtrix3 [60] with supporting FSL FMRIB [61]. Processing included denoising [62], Gibbs-ringing removal [63], distortions topup, eddy-current and motion correction with outlier replacement [64, 65], and bias-field (B1) inhomogeneity correction in ANTs [66]. Diffusion tensors were fitted using weighted least squares [67] to derive fractional anisotropy (FA), mean diffusivity (MD), axial diffusivity (AD), and radial diffusivity (RD). Tensor-based registration and tract-based spatial statistics (TBSS) were applied [68], generating a mean FA skeleton (threshold FA > 0.2) onto which individual diffusivity metrics were projected. Quality control comprised eddy-tool motion/outlier metrics, signal-to-noise assessment, and visual inspection of aligned FA maps and skeleton correspondence.

Functional images were processed in SPM [69], including slice-timing correction, realignment, nonlinear normalisation to MNI space, resampling to 3-mm isotropic voxels, and 5-mm FWHM Gaussian smoothing. First-level general linear models were estimated for each task, and key contrasts of interest were computed (details in Table 8). Preprocessed BOLD time series are available, enabling task-modulated connectivity analyses from the available derivatives, supporting hypothesis-driven analytical strategies. All EPIs and contrast maps underwent manual inspection for artefact, distortion, and misregistration. Resting-state data were processed using FSL FEAT [70] and ANTs [71], including motion correction, slice time correction, brain extraction, spatial smoothing (Gaussian kernel of 4 mm FWHM), and intensity normalisation. ICA-AROMA [72] was applied to remove motion- and physiology-related components. High-pass filtering (>0.008 Hz), boundary-based registration to T1-weighted images, and nonlinear normalisation to MNI space were performed. Residual white-matter and CSF signals were regressed to yield motion- and artefact-cleaned timeseries suitable for functional-connectivity analysis. Across modalities, all datasets underwent manual visual QC by trained technicians, with pass/fail ratings logged per scan type. Summary motion measures and QC flags are available to enable transparent sample definition.

**Table 8:** fMRI task details.

Task	Task details	Cognitive/Affective domain	Main behavioural outputs	Imaging outputs
Monetary Incentive Delay	The task comprised 66 ten-second trials. Each trial began with a 250 ms cue indicating the target’s side (left/right) and possible points (0, 2, or 10). After a 4000–4500 ms fixation, participants pressed a button corresponding to the target’s location. Feedback on points earned was shown for 1450 ms. A tracking algorithm adjusted target duration (100–300 ms) per participant to achieve ~66% correct responses.	Reward anticipation & outcome	Response time, accuracy, reward sensitivity	First-level SPM contrast maps (anticipation, outcome); preprocessed BOLD time series (fMRIPrep/Halfpipe); task-modulated connectivity derivable from task time series
Stop Signal Task	The stop-signal task (SST) included Go and unpredictable Stop trials. In Go trials, participants responded to left/right arrows with the corresponding button. In Stop trials, arrows turned upward (~300 ms later), requiring response inhibition. A tracking algorithm adjusted the Go–Stop interval to achieve ~50% successful inhibition. Inter-trial interval was 1800 ms.	Response inhibition	Stop Signal Reaction Time (SSRT), Go Response time, accuracy	First-level SPM contrast maps (Stop vs Go, successful inhibition); preprocessed BOLD time series (fMRIPrep/Halfpipe); task-modulated connectivity derivable from task time series
Emotional Faces Task	Participants viewed 18-second blocks of faces (emotions) or control stimuli. Faces showed 200–500 ms black-and-white clips of six people (three female). Nine control blocks displayed concentric circles matched for contrast and motion.	Socio-affective processing	Response time, accuracy, threat bias	First-level SPM contrast maps (emotion vs control); preprocessed BOLD time series (fMRIPrep/Halfpipe); task-modulated connectivity derivable from task time series
Resting-state fMRI	Participants were instructed to close eyes and to not focus on a specific thought (5–6 min duration).	Intrinsic connectivity	—	Whole brain functional connectivity matrices derivable from parcellated time series; network-level analyses supported

## Data management and sharing

The STRATIFY and ESTRA studies operate under a comprehensive Data Management Plan aligned with the FAIR principles (Findable, Accessible, Interoperable, Reusable) principles, to ensure long-term utility and transparency of the generated data [73]. Data handling and storage procedures were designed to maximise reproducibility and facilitate secure data sharing. A centralised, secure study database was implemented to house all data streams, including raw, processed, and derived variables. To streamline data integration, an automated upload system was developed for direct transfer from collection sites to the central database. Data quality control was ensured through strict adherence to pre-specified Standard Operating Procedures (SOPs), harmonised across recruitment sites, and applied uniformly throughout all stages of data acquisition and processing.

Data sharing is supported through an anonymised databank infrastructure developed within the IMAGEN project [50, 74], enabling sustained and secure access for the broader research community. Given the sensitive nature of the clinical, neuroimaging, genetic, and multi-omics data, access is provided via a transparent controlled-access framework rather than unrestricted public release. Access is open to qualified academic researchers, including external investigators, in accordance with participant consent, ethical approvals, and data protection regulations. Researchers seeking access must submit a proposal outlining the scientific question, requested variables, planned analyses and publication intentions. Applications are reviewed by the Executive Committee for scientific merit, feasibility, potential overlap with ongoing approved projects and compliance with ethical and legal requirements. Approved users are granted access to anonymised data via secure server (SFTP), with a data use agreement required where applicable to ensure compliance with data protection standards (GDPR). Requests should be directed to pons-centre@charite.de. Further information on the data access policy and application procedure is publicly available at www.stratify-project.org.

## Key findings

The STRATIFY and ESTRA studies have already yielded important insights into the neurobiology of psychiatric disorders, highlighting both shared and disorder-specific mechanisms and validating markers identified in the IMAGEN population-based cohort. Selected findings include:

Shared Neural Substrate of Psychiatric Comorbidity: Xie et al. (2023) [75] identified a neuropsychopathological (NP) factor derived from multi-task functional connectivity in IMAGEN, which captured transdiagnostic vulnerability across internalising and externalising dimensions. This factor persisted into young adulthood and was validated in STRATIFY and ESTRA, effectively distinguishing patients with MDD, AUD, AN, and BN from healthy controls. These results underscore the potential of network-based biomarkers for cross-disorder stratification.

Mental Health Trajectories During COVID-19: Lu. et al. (2023) [76] showed that while the STRATIFY/ESTRA clinical samples consistently exhibited higher symptom levels than the IMAGEN population cohort, trajectories diverged: depressive symptoms rose and eating disorder symptoms decreased in IMAGEN, whereas high symptom burdens persisted in clinical participants. Alcohol use declined in both. These findings demonstrate the importance of pre-existing vulnerability in shaping responses to global stressors.

Machine learning models for diagnosis and risk prediction: Zhang et al. [77] applied machine learning to multi-domain data, successfully predicting diagnoses of MDD, AUD, and EDs in STRATIFY and ESTRA, achieving areas under the curve (AUCs) between 0.80 to 0.92 for case-control classification. Shared predictors included neuroticism, hopelessness, and ADHD symptoms, pointing to overlapping risk profiles. In IMAGEN, predictive models showed moderate accuracy for the onset of ED and depressive symptoms and harmful drinking over 2–5 years (AUCs 0.64-0.71), demonstrating translational potential.

Together, these studies illustrate the translational value of linking population-based and clinical cohorts, providing a foundation for the development of predictive, biologically informed classification frameworks.

## Strengths and limitations

The STRATIFY/ESTRA studies exhibit numerous strengths. First, they provide a deeply phenotyped, harmonised multimodal dataset integrating neuroimaging, genomics, environmental exposures, cognitive measures, and biospecimens, enabling mechanistic analyses across biological scales. Second, the inclusion of multiple diagnostic groups together with well-matched healthy reference samples supports both transdiagnostic modelling and the identification of disorder-specific signatures. Third, advanced analytical frameworks, including machine learning and normative modelling, allow latent neurobehavioral dimensions to be derived from high-dimensional data, facilitating prediction of clinical outcomes and risk trajectories. Fourth, the studies are embedded within a broader European data ecosystem. The environMENTAL study [78], for example, links participants to geospatial measures of climate, pollution, green space, and urbanicity, enabling rigorous investigation of how environmental exposures interact with brain and molecular phenotypes to shape mental health risk. Crucially, STRATIFY and ESTRA are harmonised with the longitudinal, population-based IMAGEN cohort, providing a large normative reference spanning adolescence into early adulthood. Finally, extensive biobanking of blood and urine supports genome-wide genotyping, DNA methylation profiling, transcriptomics, and proteomics, enabling direct investigation of molecular pathways underlying vulnerability, resilience, and disease progression.

Several limitations should also be noted. First, the sample includes a large proportion of participants self-identifying as White/European, which may limit generalisability to more ethnically diverse populations. Future analyses using the available genetic data should model ancestry-related structure directly, for example through genetic principal components, and further studies in more diverse samples will be important to establish the generalisability of the present findings. Second, females are over-represented, particularly in the eating disorder cohorts, constraining the extrapolation of findings to males. Third, case identification for MDD, AUD and ED relied on validated questionnaire thresholds (PHQ-9, AUDIT, EDDS) rather than structured diagnostic interviews. While these instruments have good diagnostic accuracy [44, 79–83] and are widely used in large-scale studies, this approach inevitably reduces diagnostic certainty. Recovered AN and BN participants were instead assessed with the SCID-5-RV to ensure rigorous confirmation of remission; while this introduces a methodological difference between current and recovered ED groups, both instruments operationalise DSM-5 criteria, supporting conceptual comparability of diagnostic constructs across groups. Extensive multimodal phenotyping further mitigates risk by capturing clinical severity and comorbidity across groups. Finally, the psychosis group is relatively small, reducing statistical power to detect psychosis-specific effects. As such, analyses in this subgroup should be considered exploratory pending replication in larger samples or meta-analytic frameworks.

## Conclusion

STRATIFY and ESTRA represent a major step forward in the effort to develop biologically informed, transdiagnostic models of psychiatric illness. By integrating environmental, genetic, and neural data, these studies offer the potential to move beyond categorical diagnoses toward a stratified understanding of mental disorders. Their findings could help define neurobehavioral endpoints for clinical trials and support the development of targeted, personalised interventions. Ultimately, the insights gained may contribute to more effective prevention and treatment strategies, reducing the public health burden of mental illness.
